# Impact of keratoconus stage on outcome after corneal crosslinking

**DOI:** 10.1186/s12886-022-02425-8

**Published:** 2022-05-06

**Authors:** Caroline Julia Gassel, Daniel Röck, Eva-Maria Konrad, Gunnar Blumenstock, Karl Ulrich Bartz-Schmidt, Tobias Röck

**Affiliations:** 1 Centre for Ophthalmology, University Eye Hospital, Eberhard Karls University Tübingen, Elfriede-Aulhorn-Str. 7, 72076 Tübingen, Germany; 2 Institute for Clinical Epidemiology and Applied Biometry, Eberhard Karls University Tübingen, Silcherstr. 5, 72076 Tübingen, Germany

**Keywords:** Keratoconus, Crosslinking, Krumeich, CXL, Corneal crosslinking

## Abstract

**Background:**

This study aimed to analyze if the outcome after corneal crosslinking (CXL) in progressive keratoconus patients depends on the stage at which the procedure is performed. This knowledge would help to improve success of CXL and to define surgery indications in those patients.

**Methods:**

In this retrospective study, 124 consecutive eyes of 100 patients with progressive keratoconus undergoing corneal CXL at the University Eye Hospital Tübingen were included. The eyes were graded according to modified Krumeich stages depending on induced myopia or astigmatism, corneal radii, minimum corneal thickness, and morphological changes. The observation period covered November 2008 to September 2018. Preoperatively, 12 and 24 months after CXL, the best corrected visual acuity (BCVA) was determined and astigmatism as well as tomographic parameters (Kmax, Kmin, central corneal thickness (CCT), minimum corneal thickness (MCT)) were measured by means of a Scheimpflug camera system.

**Results:**

BCVA results showed significant differences between the modified Krumeich stages at 12 months (*p* = 0.014) and at 24 months postoperatively (*p* = 0.032). Also, astigmatism differed significantly among the stages at 24 months after CXL (*p* = 0.023). However, no significant differences regarding astigmatism were detectable after 12 months. In terms of Kmax, Kmin, CCT, and MCT, no significant differences between the Krumeich stages were observed.

**Conclusions:**

BCVA showed a significantly higher improvement after CXL in the early stage of keratoconus compared to a higher stage. However, the postinterventional tomographic values did not differ significantly between the different modified Krumeich stages. The significantly higher improvement in BCVA after CXL in the early stage might indicate that earlier intervention provides a higher subjective benefit to the individual. Further studies with larger sample sizes are needed to confirm these findings.

## Background

Stages of keratoconus can be categorized based on clinical characteristics using a classification Krumeich established almost a quarter of a century ago [1, 2]. Krumeich differentiates four stages of keratoconus. Therapeutic management differs depending on the stage of keratoconus. In early stages, rigid gas permeable contact lenses are used to correct vision impairment [3]. In more advanced stages with topographically established progression, corneal surgery or corneal crosslinking (CXL) are required. CXL, which was recognized as a statutory health insurance benefit in Germany by the Federal Joint Committee in mid-2018, is an established and safe treatment option for progressive keratoconus. Penetrating keratoplasty and deep anterior lamellar keratoplasty (DALK) are common methods but can pose many potential complications [4]. If stage IV of the Krumeich classification system is reached, corneal scars exist and CXL is no longer an appropriate treatment option. In these cases, penetrating keratoplasty or DALK are the methods of choice [5, 6].

Ultraviolet corneal CXL is a newer and less invasive procedure. In 1997, it was first demonstrated that the combination of riboflavin and ultraviolet (UV) irradiation is able to alter the biomechanical properties of the cornea [7]. UV-A light and riboflavin are applied to induce a stiffening of the cornea. Riboflavin serves as a photosensitizer in this process. When exposed to UV-A light, riboflavin produces reactive oxygen species inducing covalent bonds between collagen molecules and proteoglycans [8].

Overall, CXL has a good safety profile. Complications are rare compared to keratoplasty. They include corneal haze, scarring, postoperative infections or ulcers, endothelial damage, reactivation of herpes viridae infections and sterile corneal stromal infiltrates [9, 10].

In the beginning of the twenty-first century, keratoconus was a common indication for penetrating keratoplasty. Our study group reported in 2018 that keratoconus was the most common surgical indication for corneal transplantation at the University Eye Hospital in Tübingen from 2004 to 2009. However, after introduction of CXL in 2008, the need of corneal transplants in keratoconus patients decreased significantly [11]. Other studies reported similar changes in keratoplasty indications after implementation of CXL [12, 13].

CXL was shown to be effective in the improvement of visual acuity and tomographic parameters in the long term [14, 15]. Nevertheless, there are no specific guidelines regarding the ideal timing for CXL implementation. Therefore, this study was designed to investigate whether the success of CXL depends on the Krumeich stage of keratoconus at which it is performed.

To the best of our knowledge, a study evaluating the influence of the stages of keratoconus on the results of CXL has never been undertaken before.

## Methods

### Study design

In this retrospective study, 124 consecutive eyes of 100 patients were included between November 2008 and September 2018. The inclusion criteria comprised progression of the keratoconus, primary CXL treatment and a preoperative follow-up period of at least 12 months. Progression was defined as the following changes within 1 year: increase in maximum anterior sagittal curvature (Kmax) by > 1 dpt and/or decrease in minimum corneal thickness by ≥5%. Eyes with previous trauma or corneal procedures were excluded.

CXL was performed according to a slightly modified Dresden protocol as previously described [14].

The eyes were categorized based on modified Krumeich stages. We adjusted the Krumeich grading system and differentiated three different groups: group I, group II and group III. Our modified classification system is mainly based on Kmax. Group I includes the patients with Kmax < 48 dpt, group II those with Kmax = 48–53 dpt and group III those with Kmax > 53 dpt, respectively. The defining parameters of the modified grades are presented in Table 1.

**Table 1:** Definition of the groups investigated in this study

Stage	Kmax	Morphological characteristics
Group I	< 48 dpt	Vogt’s striae, no corneal scars, corneal thickness ≥ 400 μm
Group II	48–53 dpt	No central scars, corneal thickness ≥ 400 μm
Group III	> 53 dpt	No central scars, corneal thickness ≥ 400 μm

Modified Krumeich classification system of keratoconus stages defined by the presented parameters

Best corrected visual acuity (BCVA) was determined in logMAR before and after CXL by means of refractometry with additional subjective comparison. The patients did not wear rigid contact lenses during visual acuity testing. A camera system based on Scheimpflug’s principle (Orbscan II, Bausch&Lomb, Rochester, NY, USA) was used to obtain the corneal tomographic data (Kmax, Kmin, astigmatism, minimum corneal thickness, central corneal thickness). The measurements took place preoperatively and 12 as well as 24 months postoperatively.

All methods have been performed in accordance with the Declaration of Helsinki. The study was approved by the Ethics Committee of the medical faculty of Eberhard Karls University of Tübingen and the University Hospital of Tübingen (740/2018BO2).

### Statistical analysis

Quantitative measurement data are reported with the mean and standard deviation. Additionally, measured data are visualised using grouped bar charts with time of observation (preoperative, 12 and 24 months of follow-up, respectively) as the primary, and modified Krumeich stages as the secondary variable. To compare CXL outcome across modified Krumeich classes at 12 and at 24 months, respectively, analysis of covariance (ANCOVA) was performed with baseline values as covariate. Thus, the differences between the groups in each time point are adjusted for baseline values. The level of statistical significance was predefined at α = 0.05. If applicable, pairwise post-hoc comparisons of effects were carried out using the Tukey-Kramer HSD test with adjustment for multiplicity. For all statistical analyses, the JMP® 14.2 statistical software was used (SAS Institute Inc., Cary, NC, USA).

## Results

### Patient population

Regarding the gender distribution, 110 of the 124 study eyes were from males and 14 were from females. On average, the patients were 23.0 ± 8.3 years old.

### Significant differences in visual acuity and astigmatism after crosslinking according to modified Krumeich stages

The preoperative BCVA was 0.16 ± 0.17 logMAR in group I, 0.26 ± 0.23 logMAR in group II and 0.36 ± 0.14 in group III. Twelve months following CXL, BCVA in group I was 0.1 ± 0.12 logMAR, 0.23 ± 0.17 logMAR in group II and 0.25 ± 0.11 log MAR in group III. After 24 months, BCVA was 0.1 ± 0.14 logMAR in group I, 0.26 ± 0.24 logMAR in group II and 0.26 ± 0.12 logMAR in group III. In terms of BCVA, significant differences between groups in the 12 months postoperative (post-op) group (*p* = 0.014) as well as in the 24 months post-op group (*p* = 0.032) were observed compared to the preoperative assessment. The patients of group II had a significantly lower increase in visual acuity than those of group I (group II vs. I: *p* = 0.011 at 12 months post-op; *p* = 0.03 at 24 months postop).

Patients of group I presented with a mean astigmatism of 3.04 ± 1.7 dpt. In group II, the mean astigmatism was 4.43 ± 2.08 dpt, in group III 6.67 ± 3.48 dpt. Twelve months post-op, astigmatisms of 2.98 ± 1.61 dpt in group I, 4.02 ± 2.07 dpt in group II and 5.77 ± 3.1 dpt in group III were measured. After 24 months, the astigmatism was 3.08 ± 1.38 dpt in group I, 3.82 ± 2.01 dpt and 5.69 ± 2.69 dpt in groups II and III, respectively. Regarding astigmatism, results of the 12 months post-op patients did not differ significantly among the three groups (*p* = 0.39). However, after 24 months, significant differences between the three groups were detectable (*p* = 0.023). Especially group II achieved a significant improvement regarding astigmatism compared to group I (group II vs. I: *p* = 0.003 at 24 months post-op).

### No significant impact of the modified Krumeich stages on tomographic parameters

Preoperatively, average Kmax in group I was 45.4 ± 2 dpt, and 50.3 ± 1.5 dpt and 55.2 ± 1.5 dpt in groups II and III, respectively. At 12 months following CXL, mean Kmax was measured 45.3 ± 2.1 dpt in group I, 49.2 ± 1.9 dpt in group II and 53.8 ± 2.1 in group III. At 24 months post-intervention, the average Kmax was measured 45.2 ± 2.2 dpt in group I, 48.2 ± 3.5 dpt in group II and 53.9 ± 1.7 dpt in group III.

Measured Kmax values did not differ significantly between the three groups 12 months (*p* = 0.08) or 24 months (*p* = 0.27) after surgery.

Regarding Kmin, preoperative average values were 42.4 ± 1.7 dpt in group I, 45.7 ± 1.8 dpt in group II and 48.6 ± 3.3 dpt in group III. One year after the procedure, Kmin was 42.3 ± 1.8 dpt in group I, 45 ± 2.3 dpt in group II and 48 ± 2.7 dpt in group III. Two years following CXL, Kmin was 42.3 ± 2 dpt, 44.5 ± 3.7 dpt and 48.3 ± 2.6 dpt in the groups I, II and III.

The postoperative Kmin results also showed no significant alterations between the modified stages (12 months post-op: *p* = 0.47; 24 months post-op: *p* = 0.65).

Before CXL, mean MCT was 483.5 ± 30.7 μm in group I. Groups II and III presented with a mean MCT of 468.2 ± 31.1 μm and 450.5 ± 36.5 μm before the procedure. 12 months after CXL, mean MCT was measured lower at 472.8 ± 38.7 μm, 472.8 ± 38.7 μm and 432 ± 41.4 μm in each group. After 2 years, the average MCT was 477.9 ± 43.7 μm, 446.2 ± 52.1 μm and 429.4 ± 47 μm in groups I-III.

No significant differences in minimum corneal thickness were noticed after 12 (*p* = 0.32) or 24 months (*p* = 0.39) depending on the groups.

Before CXL, mean CCT was 511.3 ± 30.8 μm in group I, 491.5 ± 25.7 μm in group II and 479.9 ± 35.6 μm in group III. Twelve months after the intervention, CCT values were lower, at 500.6 ± 34.7 mm in group I, 470.9 ± 43.8 μm in group II and 463.8 ± 42.3 μm in group III. After 24 months, 501.9 ± 39.4 μm in group I, 476.6 ± 42.9 μm in group II and 461.4 ± 36 μm in group III were measured.

Similar to the previous results, the CCT did not differ significantly between the different Krumeich stages 12 months (*p* = 0.24) or 24 months (*p* = 0.60) post-op.

**Fig. 1:**
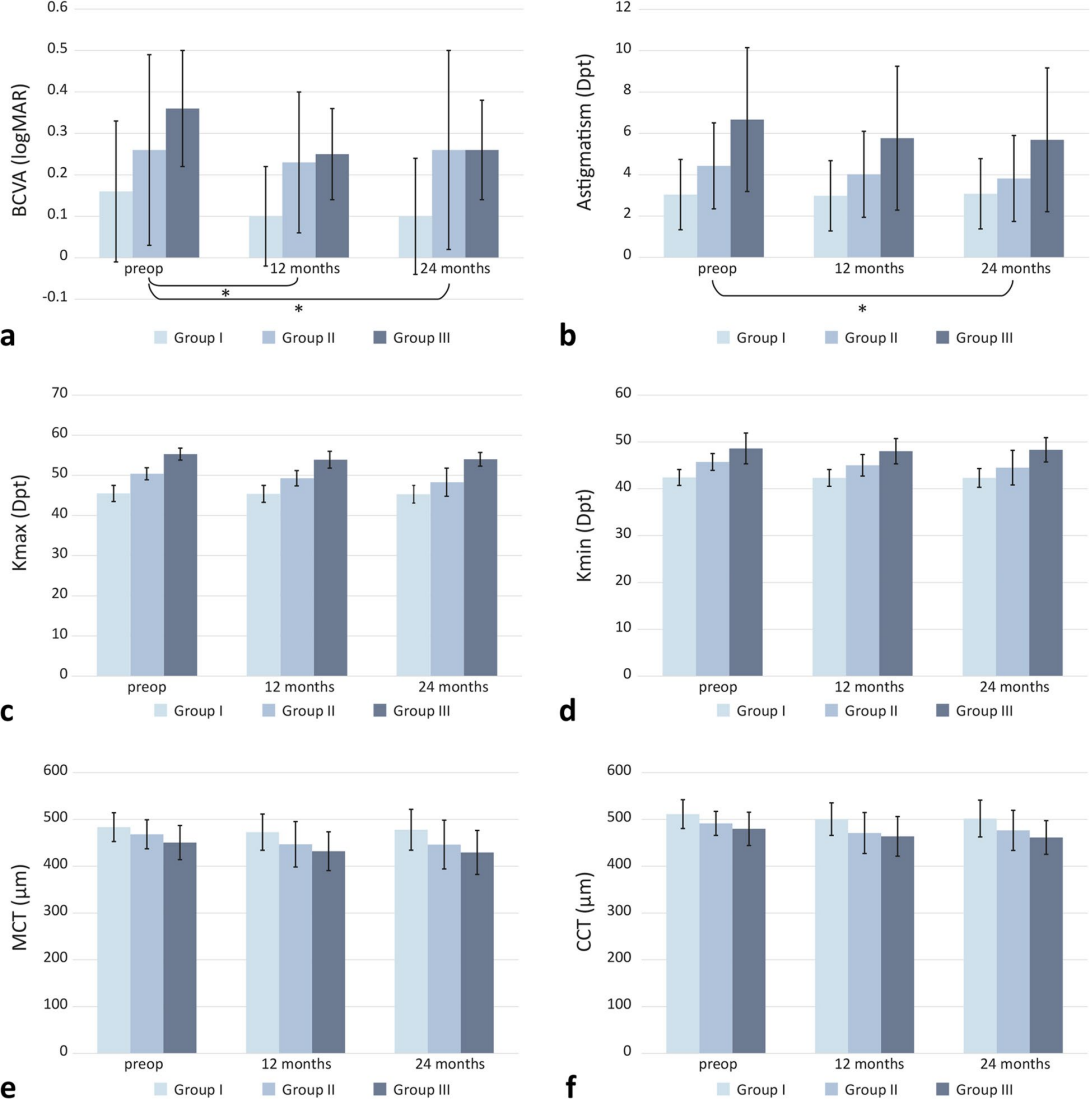
BCVA and tomographic parameters depending on the three groups. Data presented as mean ± SD preoperatively and 12 and 24 months following corneal CXL. **a**) BCVA, **b**) astigmatism, **c**) Kmax, **d**) Kmin, **e**) MCT, **f**) CCT. Statistically significant group differences (*p < 0.05) were observed for BCVA (12 months and 24 months post-op) and astigmatism (24 months post-op)

In Fig. 1 and Table 2, the respective BCVA and tomographic values at the respective measurement times in the three groups are presented.

**Table 2:** BCVA, astigmatism and tomographic values of the three groups preoperatively and after 12 and 24 months

	preop			12 months			***P*** value 12 months	24 months			***P*** value 24 months
Group	I	II	III	I	II	III		I	II	III	
**BCVA**	0.16 ± 0.17	0.26 ± 0.23	0.36 ± 0.14	0.1 ± 0.12	0.23 ± 0.17	0.25 ± 0.11	**0.014**	0.1 ± 0.14	0.26 ± 0.24	0.26 ± 0.12	**0.032**
**Astigm**	3.04 ± 1.7	4.43 ± 2.08	6.67 ± 3.48	2.98 ± 1.61	4.02 ± 2.07	5.77 ± 3.1	0.39	3.08 ± 1.38	3.82 ± 2.01	5.69 ± 2.69	**0.023**
**Kmax**	45.4 ± 2	50.3 ± 1.5	55.2 ± 1.5	45.3 ± 2.1	49.2 ± 1.9	53.8 ± 2.1	0.08	45.2 ± 2.2	48.2 ± 3.5	53.9 ± 1.7	0.27
**Kmin**	42.4 ± 1.7	45.7 ± 1.8	48.6 ± 3.3	42.3 ± 1.8	45 ± 2.3	48 ± 2.7	0.47	42.3 ± 2	44.5 ± 3.7	48.3 ± 2.6	0.65
**MCT**	483.5 ± 30.7	468.2 ± 31.1	450.5 ± 36.5	472.8 ± 38.7	446.8 ± 48.6	432 ± 41.4	0.32	477.9 ± 43.7	446.2 ± 52.1	429.4 ± 47	0.39
**CCT**	511.3 ± 30.8	491.5 ± 25.7	479.9 ± 35.6	500.6 ± 34.7	470.9 ± 43.8	463.8 ± 42.3	0.24	501.9 ± 39.4	476.6 ± 42.9	461.4 ± 36	0.60

The means, standard deviations and *p* values of the respective measurement data at the respective times in the different groups are presented. Bold font indicates statistical significance

*BCVA* Best corrected visual acuity, *Astigm* Astigmatism, *Kmax* Maximum corneal curvature, *Kmin* Minimum corneal curvature, minimum corneal thickness, *MCT* Minimum corneal thickness, *CCT* Central corneal thickness

## Discussion

Previous studies of our working group demonstrated a significant reduction of tomographic values and a significant increase in BCVA in a long-term follow-up after corneal CXL (14,15). Usually, CXL is performed if the keratoconus progression parameters worsen within 1 year. These parameters include the reduction of the minimum corneal thickness by at least 5% or an increase of Kmax by one dioptre [16]. However, there is no consensus about the best point in time to indicate the procedure. This raises the question if the pathomorphological stage, which can be defined by the Krumeich classification, has an influence on the postinterventional outcome. In the clinical routine, it is common for patients to ask whether it is better to have CXL performed sooner or later. So far, science has not been able to provide an answer to this important question. But this knowledge could help to determine the best time to indicate corneal CXL. By now, there is no literature about the best outcome of CXL depending on the Krumeich stages. This study is the first to investigate this relationship.

Interestingly, this study did not observe any significant differences between the modified Krumeich stages in terms of tomographic values. Nevertheless, BCVA and astigmatism differed significantly depending on the modified Krumeich stages. The patients in group I had a significantly greater improvement in BCVA than those in group II. This could imply that patients subjectively benefit more from earlier intervention.

It is conceivable that there is a relationship between the influence of astigmatism and BCVA after CXL. However, it is unclear why the keratoconus stages have no significant impact on the improvement of tomographic parameters following the procedure. This would mean that CXL seems to be similarly effective in terms of tomographic parameters in each of the three modified Krumeich stages which we investigated in this study. Nevertheless, some points should be considered before drawing rash conclusions.

The limitations of this study comprise the retrospective design with a possible selection bias and the impossibility to determine causation. Retrospective analyses are subject to confounding, which could potentially bias the results. Nonetheless, our data are supported by a relatively large case number of 124 eyes. To further investigate the impact of the keratoconus stage on post-CXL outcome, a large controlled prospective study should be performed. Another limitation that should be considered is the postoperative follow-up time of 24 months which might be too short to detect the true significant effects in the three different groups of this study. Recently, Vinciguerra et al. reported a study with a follow-up time after CXL up to 13 years [17]. Tasci et al. retrospectively analysed long-term visual acuity, topographic and aberrometry results in a period up to 5 years after CXL. They concluded that CXL improves visual acuity and quality of vision by reducing higher-order aberrations and spherical aberrations, and stops the progression of keratoconus [18]. However, they did not investigate which factors had a particularly positive or negative impact on the postinterventional results.

We plan to observe our patients 10 years after CXL in the future to gain further knowledge.

It may also be of interest to examine other factors which could influence the postinterventional results and therefore might play a role in the indication for CXL. For example, the patients’ age or the actual rate of keratoconus progression may have an impact on the outcome. In a prospective comparative case series from Egypt, 22 keratoconus patients younger than 18 years underwent CXL and showed a significant improvement of visual acuity and tomographic values postoperatively, with no evidence of progression of keratoconus over 12 months [19]. A retrospective study of 96 eyes from Turkey detected that patients older than 30 years, patients with a worse baseline BCVA and those with a thinner baseline pachymetry benefit most likely from CXL [20].

A validation study from Godefrooij et al. demonstrated that a lower visual acuity before CXL is the sole independent factor which can predict an improvement in visual acuity 12 months after the procedure. This finding suggests that patients with a lower vision are more likely to benefit from CXL [21]. These data imply that in terms of visual acuity, higher stages of keratoconus benefit more from CXL. Since in our study we mainly used Kmax for classification into the different groups I, II and III, this does not contradict our results. A retrospective analysis from France postulates lower preoperative visual acuity, high astigmatism and advanced keratoconus as predictive factors for visual improvement after CXL [22].

In contrast to this, a retrospective analysis from Lebanon with 156 eyes and a minimum follow-up of 3 years demonstrated that a better preoperative visual acuity was associated with a higher vision improvement. A higher baseline Kmax was associated with a worse postinterventional visual acuity [23].

Overall, the literature contains very controversial results. In future, controlled prospective studies with a larger sample size should investigate the respective influence of the individual factors such as keratoconus stage, age and visual acuity. These findings could improve patient management and help define the optimal time to indicate CXL.

## Conclusion

Our data suggest that patients benefit from corneal CXL regardless of modified Krumeich stages. However, we observed trends towards a comparatively higher improvement in visual acuity in the group with the early keratoconus stage. Larger controlled studies over longer observation periods should be conducted to verify these observations. These findings may optimize the management and indication of corneal CXL in keratoconus patients.

## Data Availability

The datasets used and/or analysed during the current study are available from the corresponding author upon reasonable request.

## Abbreviations

BCVA: Best corrected visual acuity
CCT: Central corneal thickness
CXL: Corneal crosslinking
DALK: Deep anterior lamellar keratoplasty
Kmax: Maximum corneal curvature
Kmin: Minimum corneal curvature
MCT: Minimum corneal thickness
UV: Ultraviolet
